# Comparison of different soft grippers for lunch box packaging

**DOI:** 10.1186/s40638-017-0067-1

**Published:** 2017-11-02

**Authors:** Zhongkui Wang, Mingzhu Zhu, Sadao Kawamura, Shinichi Hirai

**Affiliations:** 1 Department of Robotics, Ritsumeikan University, Noji-Higashi 1-1-1, Kusatsu, 525-8577 Japan; 2 Ritsumeikan Global Innovation Research Organization, Ritsumeikan University, Noji-Higashi 1-1-1, Kusatsu, 525-8577 Japan

**Keywords:** Soft pneumatic actuator, Soft gripper, Fabrication, Grasping, Lunch box packaging

## Abstract

Automating the lunch box packaging is a challenging task due to the high deformability and large individual differences in shape and physical property of food materials. Soft robotic grippers showed potentials to perform such tasks. In this paper, we presented four pneumatic soft actuators made of different materials and different fabrication methods and compared their performances through a series of tests. We found that the actuators fabricated by 3D printing showed better linearity and less individual differences, but showed low durability compared to actuators fabricated by traditional casting process. Robotic grippers were assembled using the soft actuators, and grasping tests were performed on soft paper containers filled with food materials. Results suggested that grippers with softer actuators required lower air pressure to lift up the same weight and generated less deformation on the soft container. The actuator made of casting process with Dragon Skin 10 material lifted the most weight among different actuators.

## Introduction

In Japan, people often eat box lunches for the convenience and great varieties. Every day, several million box lunches are produced and consumed in Japan. Considering the hygiene and freshness, lunch boxes are usually manufactured and distributed locally. So far, the packaging of lunch boxes is still performed by human labors due to the fragility, variety, high deformability, and the individual differences in shape and physical property of food materials [1]. To reduce labor cost, automation systems for lunch box packaging are highly demanded in food industry.

**Fig. 1:**
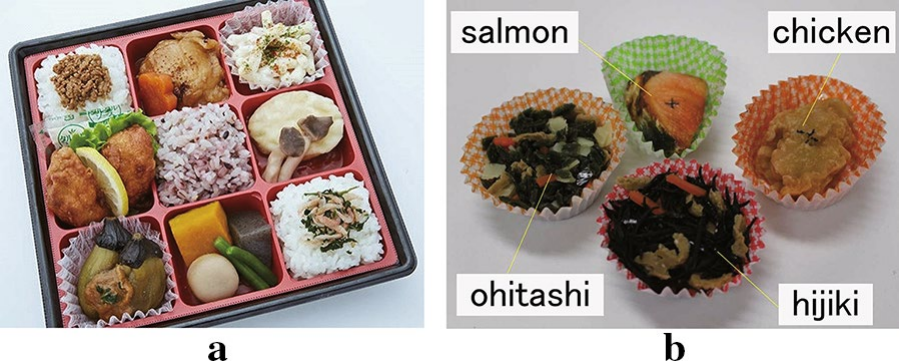
**a** A commercial box lunch and **b** typical side dishes filled in paper containers

**Fig. 2:**
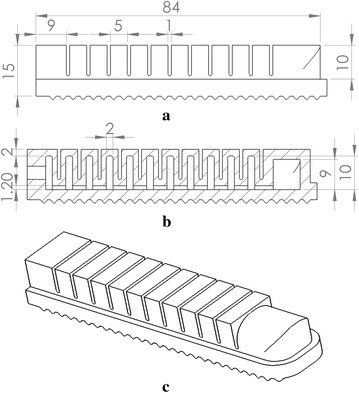
The actuator design: **a** the front view, **b** the section view, and **c** the isometric view

A typical lunch box (Fig. 1a) usually consists of rice and dishes distributed in soft paper containers. The paper containers (Fig. 1b) usually have a frustum shape and highly deformable. Picking and placing such containers filled with food materials is the main task for lunch box packaging. The traditional rigid grippers and vacuum packaging systems, which have been widely used in food industry, have difficulties to perform such a task because the rigid gripper may damage the food material and the vacuum system needs a flat surface to allow suction. New grasping mechanism providing gentle grasps is required to cope with this task.

In recent years, pneumatic soft robotic grippers have drawn great attention from researchers because of their flexibility and adaptability. Pioneer works in developing pneumatic soft gripper were conducted by Suzumori et al. in the 1990s of the last century. They proposed a four-fingered gripper made of fiber-reinforced rubber with three cylindrical air chambers and experimentally tested different grasping modes [2, 3]. Similar ideas were also proposed and applied in constructing flexible arm links [4–6], soft robotic glove for at-home rehabilitation [7], and a manta swimming robot [8]. Another idea for constructing pneumatic soft actuator is to use pleated chamber morphology and was firstly proposed by Ilievski et al. [9]. Marchese et al. [10] summarized the design and fabrication of soft fluidic elastomer robots and divided such robots into three types: ribbed, cylindrical, and pleated, based on their chamber morphologies. Comparing with the first two types, the pleated type is capable of bending to higher curvatures and exerting higher maximum forces because of its ability to accommodate the largest energy input. Therefore, this idea was widely used to actuate soft robots, such as the soft planar grasping manipulator [11], the soft gripper for biological sampling on deep reefs [12], and a soft gripper for object identification [13].

According to [10], the main disadvantage of pleated design is the complex fabrication process which involves several casting processes. To simplify the fabrication process, 3D printing technology has been adopted and several gripper designs have been proposed. MacCurdy et al. [14] presented a two-finger gripper using printable hydraulic technology. Peele et al. [15] proposed a 3D printable soft actuator using projection stereolithography. Most recently, Yap et al. [16] presented a high-force soft gripper fabricated using common 3D printer and fused deposition modeling (FDM) technology. This gripper is promising for handling heavy objects and it can lift a weight up to 5 kg with a maximum payload-to-weight ratio of 1805%. However, the authors concluded that this gripper is not suitable for applications where low pressure and delicate force are required due to the relatively hard material property of NinjaFlex.

In our previous work, we have presented a 3D printed soft gripper using Objet260Connex printer (Stratasys, MN, USA) [17] and integrated a curvature sensor to capture the bending behavior of the actuators [18]. We also proposed a simplified line-segment model to calculate the deformation behavior of the actuator [19]. Our previous actuator was printed as two separate parts and glued together to form seamless chambers. In this study, we presented two ways to print the actuator in one shot to further simplify the fabrication process. For comparisons, we also presented another two grippers fabricated by traditional casting procedure.

## Methods

### Design of the soft actuator

The soft actuator design is based on the idea of the pleated type morphology of the fluidic elastomer robot. As shown in Fig. 2, the actuator has a similar size of an Asian male’s finger and consists of twelve soft air chambers. Among the chambers, eleven of them have a wall thickness of 1.5 mm and one larger chamber at the end has a wall thickness of 3 mm. The thicker wall of the larger chamber makes the actuator end stiffer than the rest of the actuator to mimic the function of human nail. A 1.2-mm groove was designed to cross the bottom of all chambers to allow air passing through. A hole with a diameter of 4 mm was designed on the left-side wall to allow the insertion of the air hose. Rippled structure was designed on the bottom surface of the actuator to increase the grasping stability and mimic the human fingerprint. Performance effects of geometry variations of the soft actuator design present an important and interesting issue, but it is out of the scope of this paper, in which we are mainly focusing on the variations of material and fabrication process.

**Fig. 3:**
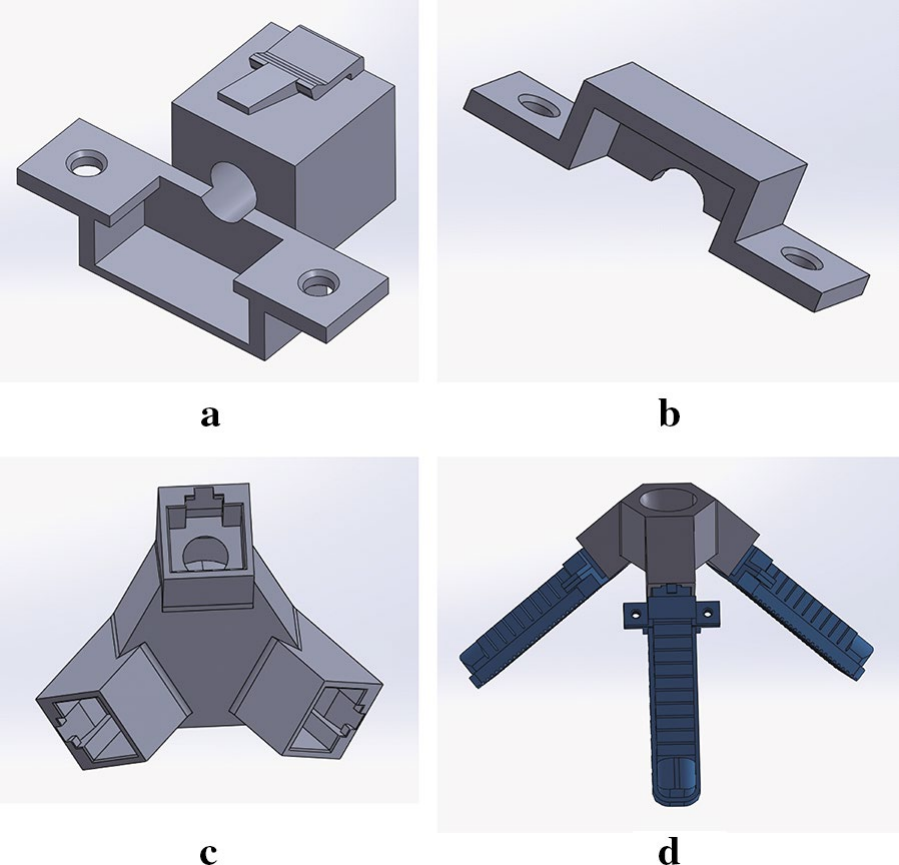
The assembly components: **a** the bottom half, **b** top half of the connector, **c** the gripper base, and **d** the assembled soft gripper

**Fig. 4:**
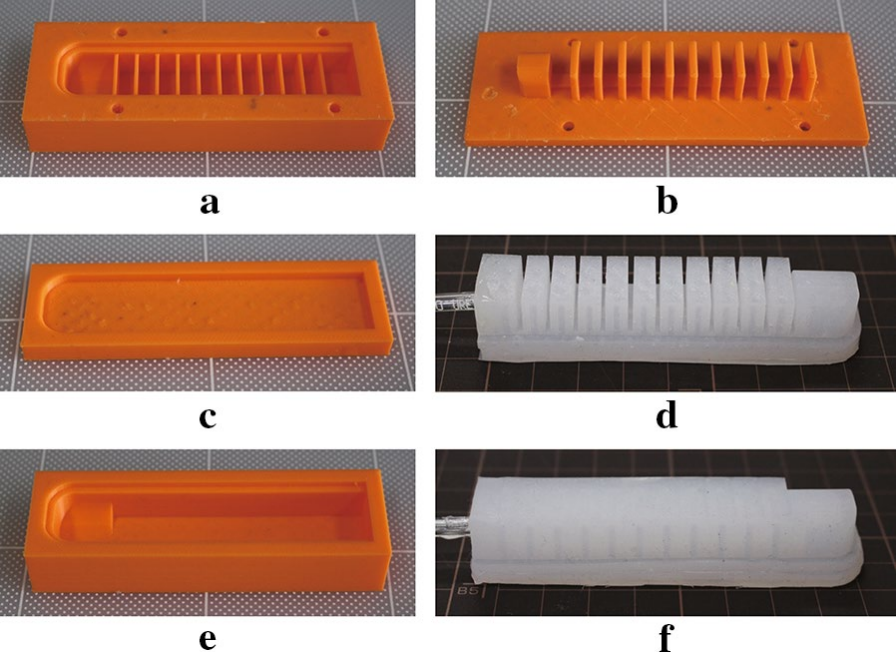
The casting process: **a** the chamber bottom mold, **b** the chamber top mold, **c** the cover mold, **d** the soft actuator with separate chambers (actuator No. 1), **e** the wrapping mold, and **f** the soft actuator with wrapped chambers (actuator No. 2)

### Design of connector and base

To connect multiple soft actuators and construct a gripper, we designed a rigid connector and a gripper base. The connector consists of two parts: the bottom half and top half as shown in Fig. 3a, b. During assembling, the soft actuator was firstly fitted into the bottom half and the air hose was inserted through the hole on the connector. Then, the top half of the connector was covered on the top of the actuator and both halves were fixed and screwed together. The cavity height formed by both halves of the connector was designed 1 mm shorter than the actuator height. Therefore, the actuator can be fixed stably after screwing together the connector. Considering the circular shape of the grasping target (the paper container in Fig. 1b), we designed a gripper base (Fig. 3c) with three female connectors distributed circularly. A snap-lock mechanism consisting of a male (on the bottom half of the connector) and a female (on the base) interfaces was designed for assembling the connector to the base without using screws. The assembly is shown in Fig. 3d. Three actuators were chosen because we believe that three is the minimum number of actuators to achieve a stable grasping and the grasping stability will be increased by using more actuators.

**Fig. 5:**
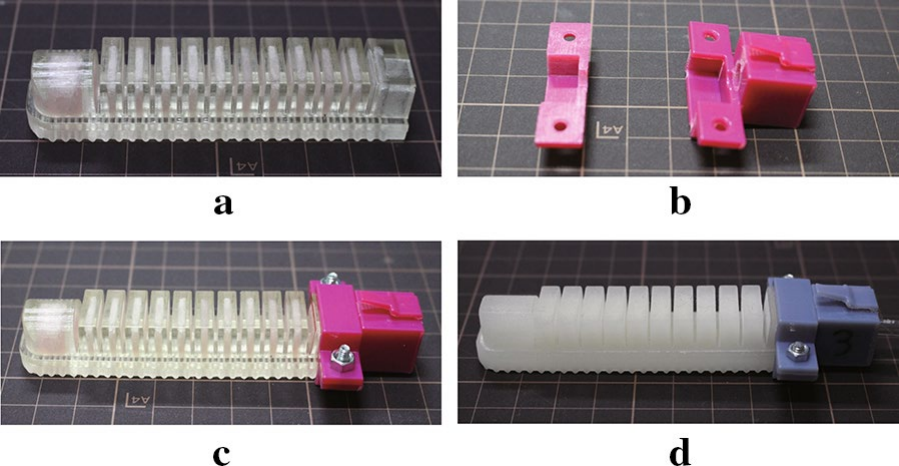
The 3D printed soft actuators: **a** soft actuator fabricated by Objet350 Connex3 (actuator No. 3), **b** the connector parts, **c** the assembled actuator, and **d** the assembled actuator which was fabricated by Agilista (actuator No. 4)

**Fig. 6:**
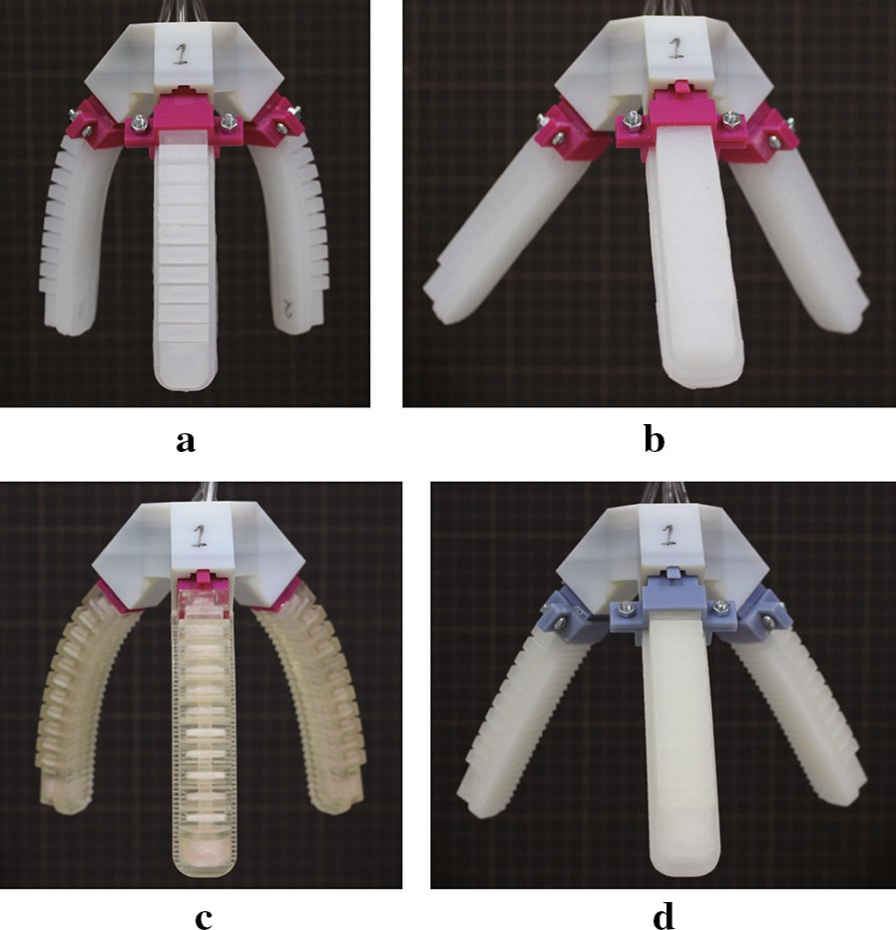
The assembled grippers: **a** No. 1, **b** No. 2, **c** No. 3, and **d** No. 4

### Actuator fabrication

Two methods and four materials were used to fabricate the soft actuators. Two methods are: (1) the traditional casting process, (2) 3D printing using the Objet350 Connex3 printer (Stratasys, Minnesota, USA) and the Agilista printer (Keyence, Japan). Four materials are: (1) the Dragon Skin 10 (Smooth-on Inc., PA, USA), (2) the Ecoflex (Smooth-on Inc., PA, USA), (3) the TangoPlus or TangoBlackPlus (Stratasys, MN, USA), which mainly consists of propenoic acid, ethyl ester, and trimethylbicyclo, and has a hardness of Shore A26-A28 and an elongation at break of 170–220%, and (4) the AR-G1L (Keyence, Japan), which mainly consists of silicone and acrylate monomer, and has a hardness of Shore A35 and an elongation at break of 160%.

#### Casting process

We fabricated molds using 3D printer Zortrax M200 (Zortrax, Olsztyn, Poland). The Dragon skin and Ecoflex materials were used to fabricate two types of soft actuators. First, we poured the Dragon skin into the chamber bottom mold (Fig. 4a) and then dipped the chamber top mold (Fig. 4b) into the bottom mold and fixed the two molds using screws. After curing, the model with open chambers was carefully removed from the molds. Second, the Dragon skin material was poured into the cover mold (Fig. 4c) to make a cover. Third, the cured chambers and cover were glued together by spreading a thin slice of Dragon skin material on both gluing surfaces. After curing, we completed the first type actuator as shown in Fig. 4d. This actuator has open spaces between neighboring chambers. Therefore, it is easy to bend and requires lower pressure to actuate. To have a stronger actuator and a closed appearance, we poured Ecoflex material into the wrapping mold (Fig. 4e) and dipped the previously completed actuator (Fig. 4d) into it. After curing and removing from the mold, we completed the second-type actuator as shown in Fig. 4f.

**Fig. 7:**
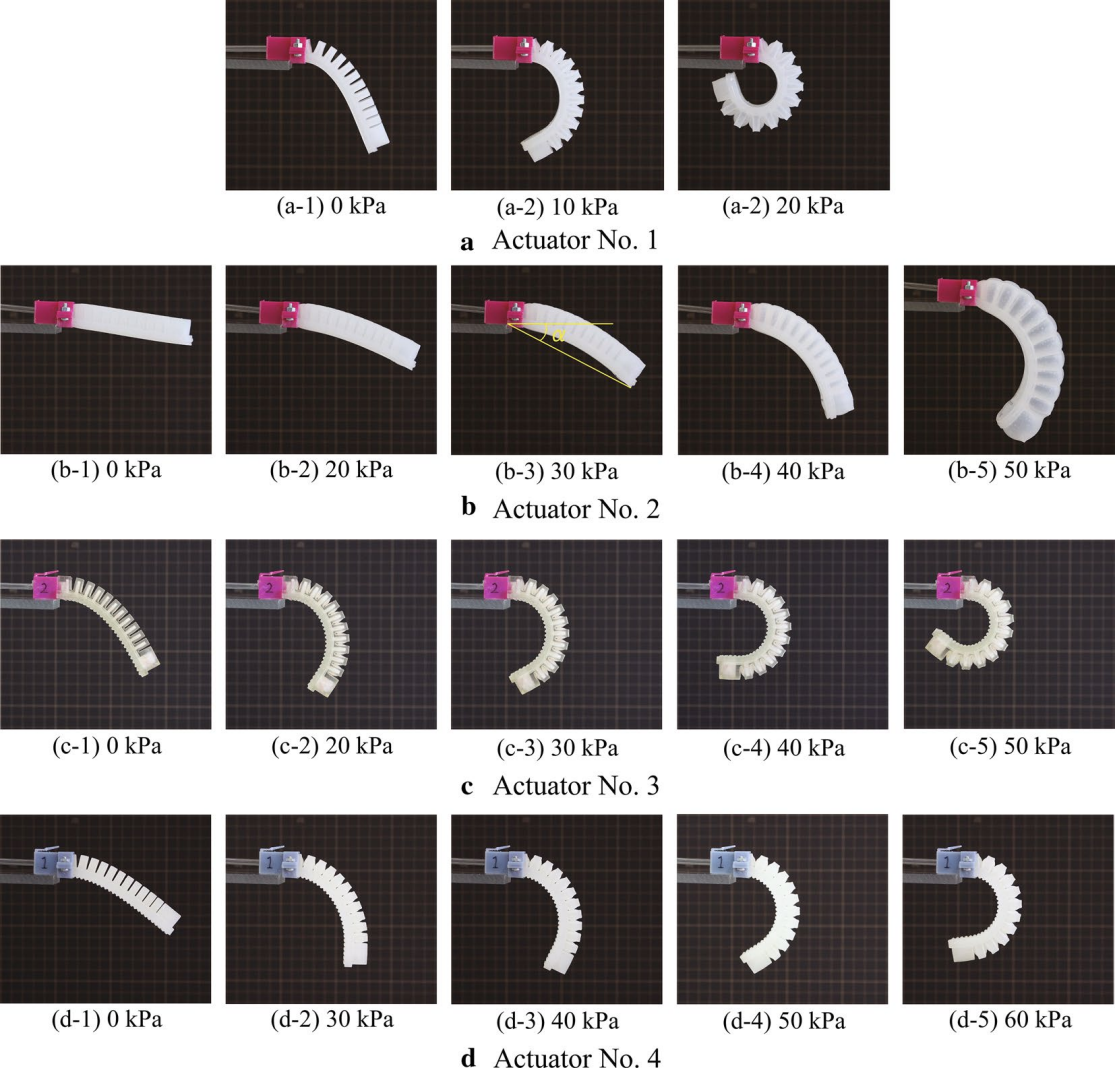
Experimental results of single actuator tests

**Fig. 8:**
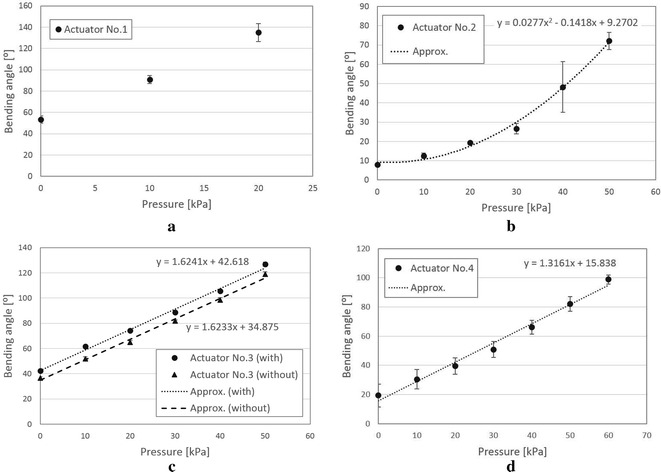
Relationship of the input air pressure and bending angle: **a** No. 1, **b** No. 2, **c** No. 3 (with and without support material), and **d** No. 4. The error bars indicate the standard deviations

**Fig. 9:**
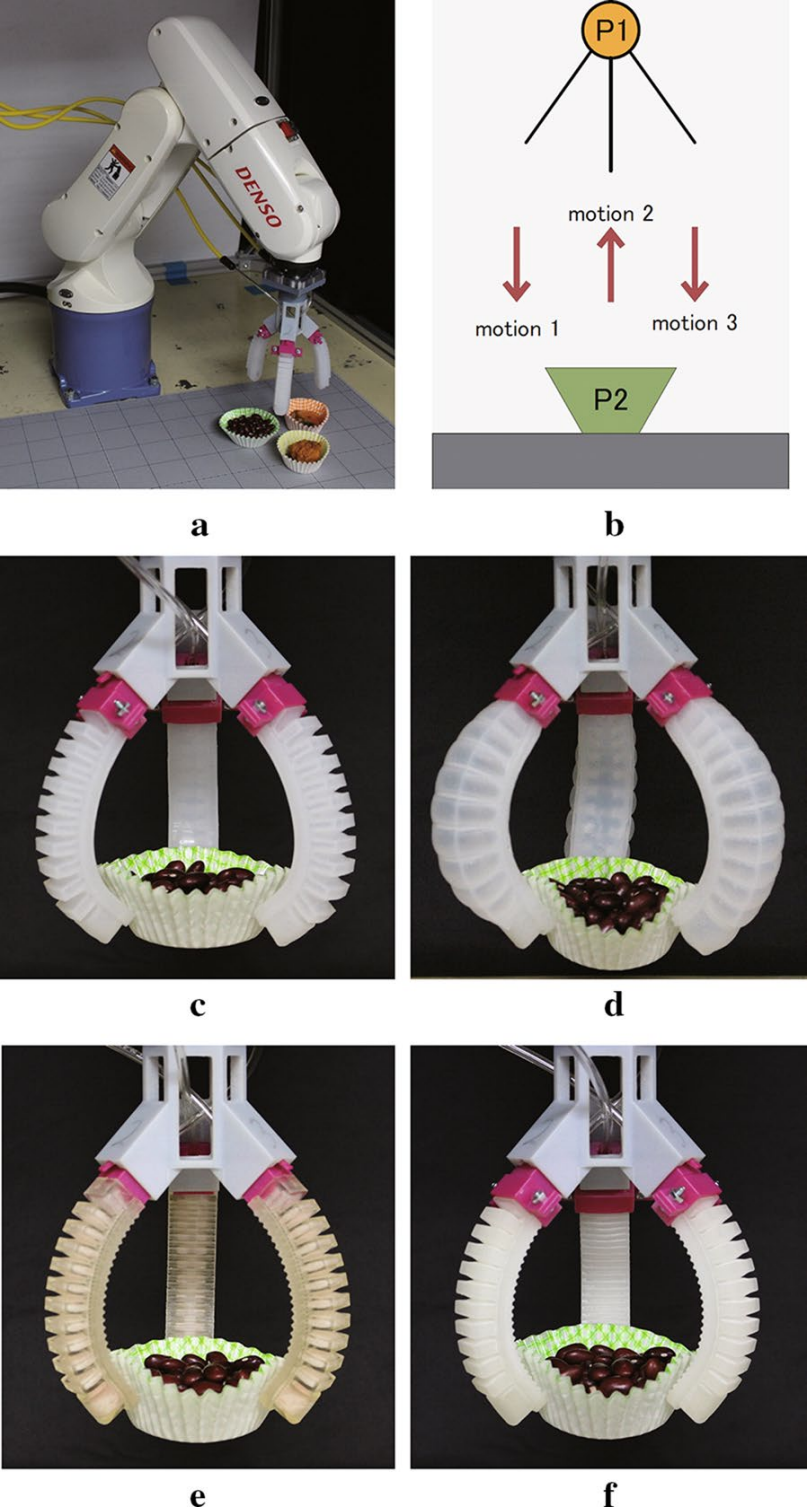
Weight grasping test: **a** experimental scenario, **b** the pick-and-place motion, and the successful grasping using gripper of: **c** No. 1, **d** No. 2, **e** No. 3, **f** No. 4 with input air pressure of 20, 50, 40, and 50 kPa, respectively

#### 3D printing

We fabricated the soft actuators (Fig. 5) using the Objet350 Connex3 and Agilista printers. It takes around one and half hours for Objet350 Connex3 and around two hours for Agilista to print the soft actuators. The connectors and base were printed by Objet350 Connex3. After printing, the actuators fabricated by Agilista were put inside water to dissolve the support materials. For the actuator fabricated by Objet350 Connex3, we removed the support material inside the groove at the bottom side (Fig. 2b) to allow air passing through. Since the support material is granular and not very sticky, we could easily separate the support material from the chamber walls by simply pressing the chambers from external surfaces. Therefore, we did not remove all the support materials from the chambers and the actuator could be inflated. In fact, the actuator responded faster with support material inside the chambers compared to the actuator with empty chambers because less air was required to inflate the chambers. After fixing the connector on the soft actuator, we assembled the gripper. To compare the performance with and without support material, we also fabricated the same actuator by Objet350 Connex3 without using support material as shown in Fig. 12a.

#### Gripper assembly

After fabricating the gripper base (Fig. 3c), we assembled the grippers (Fig. 6) using the proposed four types of soft actuators. The materials used in this study have an increasing order of hardness as: Ecoflex, Dragon skin, TangoPlus, and AR-G1L. Therefore, the gripper in Fig. 6a has the smallest initial grasping opening under gravity. The gripper with the wrapped actuators (Fig. 6b) was found to have the largest initial grasping opening because the air chambers were connected by Ecoflex material.

**Fig. 10:**
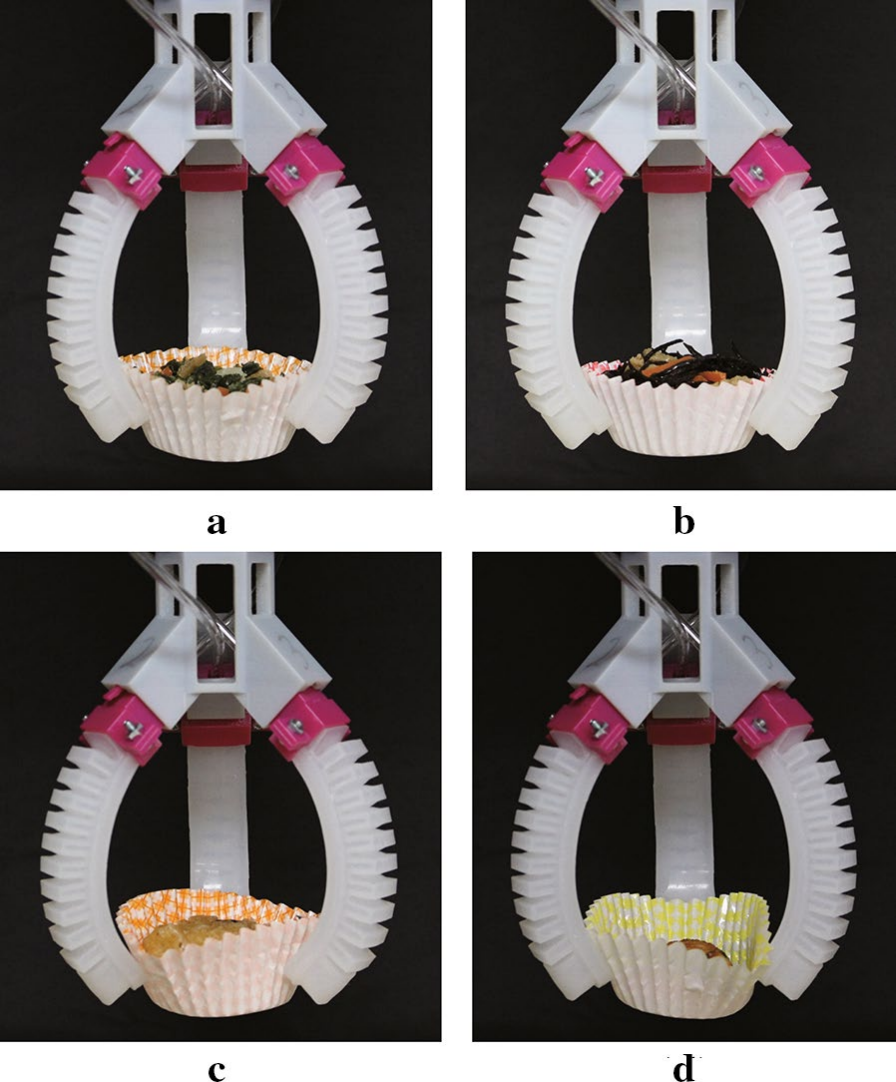
Food grasping tests: paper cups filled with: **a** ohitashi (water boiled vegetables), **b** hijiki, **c** fried chicken, and **d** salmon fish, respectively

**Fig. 11:**
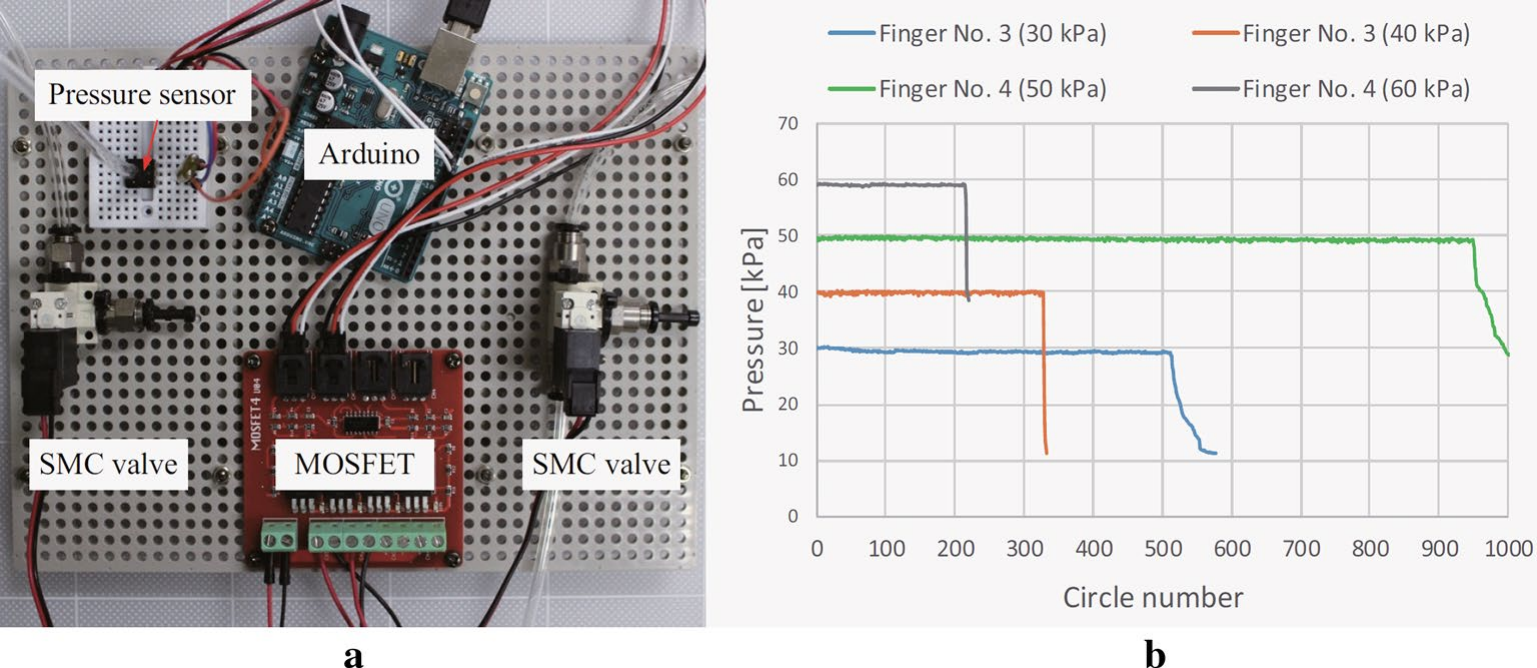
Durability test: **a** system setup, **b** test results of actuators No. 3 and No. 4

**Fig. 12:**
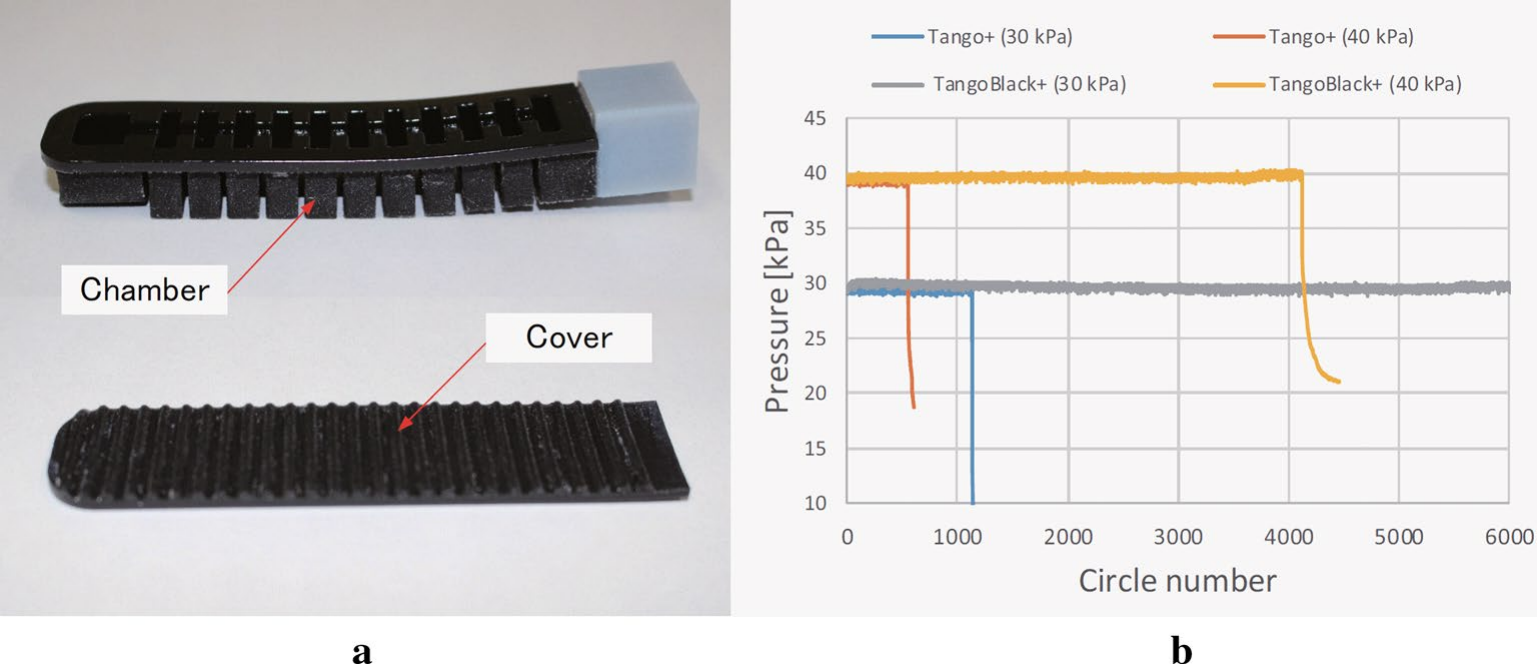
**a** Soft actuator design with two separate parts, and **b** the results of durability tests at pressures of 30 and 40 kPa for two materials of TangoPlus and TangoBlackPlus

## Results

For experimental tests, we employed an air compressor (JUN-AIR 3-4) and an electro-pneumatic regulator (SMC ITV2030) to provide constant air pressures. We experimentally tested the performance of different actuators under different air pressures and different soft grippers grasping the paper container filled with food materials.

### Single actuator test

Figure 7 shows experimental snapshots of different actuators under different air pressures. Under gravity, actuator No. 1 has the largest bending because Dragon skin is the softest comparing with TangoPlus and AR-G1L. Actuator No. 2 bent the least under gravity because the opening regions between neighboring chambers were wrapped by Ecoflex material. During experiments, the input pressure was started from 10 kPa and increased every 10 kPa until the actuator bends over $90^{\circ }$. The definition of bending angle $\alpha$ is indicated in Fig. 7(b-3). Due to the softness, the actuator No. 1 bent over $120^{\circ }$ under a pressure of 20 kPa. Instead of bending, actuator No. 2 expanded significantly along the pressure increasing and did not reach $90^{\circ }$ under a pressure of 50 kPa, after which we stopped the pressure increasing. Compared to actuator No. 3, actuator No. 4 generated less bending because the material AR-G1L is stiffer than material TangoPlus.

Three actuators for each type were fabricated and tested. Bending angles under different air pressures were calculated using ImageJ (https://imagej.nih.gov/ij/). The relationships between the input pressure and the averaged bending angle are plotted in Fig. 8 with the standard deviations indicated by the error bars. Since only three test points were available for actuator No. 1, we did not plot the approximation of the data. We found the nonlinearity and the largest individual difference in actuator No. 2. This can be explained by the two materials combination and complex manual fabrication process. The 3D printed actuators showed better linearity and less individual differences compared to casting fabrication. Apparently, actuator No. 3 fabricated by 3D printer Objet350 Connex3 has the best performance (Fig. 8c) in terms of the linearity and individual difference. The influence of support material can also be seen in Fig. 8c. Without using support material, bending angles became a little less due to the lighter weight, but the linear relationship against the input pressure was similar compared to actuator with internal support material.

### Weight grasping test

Weight grasping tests were performed using the grippers and a paper container filled with different weights of red beans. The grippers were mounted onto a commercial Denso robot arm (Fig. 9a), and a pick-and-place motion was programed. The motion was started from position 1 (P1 in Fig. 9b). Firstly, the gripper moved down (motion 1) to position 2 (P2) where the target was placed. Once arrived P2, the gripper was pressurized and attempted to grasp the target. After 5 s, the gripper was lifted up (motion 2) and back to P1, where the gripper was programmed to wait for 10 s. Finally, the gripper was brought down (motion 3) to P2 again to release the target. Ten seconds of grasping without dropping was considered as a successful test. The weight test protocol is: (1) the target weight was started from 20 g and increased every 10 g, (2) the input air pressure was started from a value where the gripper succeeded 10 times of the pick-and-place tests and increased every 10 kPa, (3) we increased the input pressure if the gripper failed to pick up a weight more than three times, (4) the tests ended at a target weight of 90 g because the container was full and 90 g is heavy enough for representing most of the side dishes in a typical Japanese lunch box.

**Table 1:** The succeeded test numbers of different grippers lifting different weights

Actuator	Pressure (kPa)	Target weights (g)							
		20	30	40	50	60	70	80	90
No. 1	10	10	7	–	–	–	–	–	–
	20	–	10	10	10	10	10	10	10
No. 2	40	10	8	5	–	–	–	–	–
	50	–	10	10	4	–	–	–	–
No. 3 (with)	30	10	10	9	0	–	–	–	–
	40	–	–	10	10	10	10	6	–
No. 3 (without)	30	10	10	10	0	–	–	–	–
	40	–	–	10	10	10	10	8	–
No. 4	50	10	10	9	10	0	–	–	–
	60	–	–	–	–	10	10	10	7

Sign “–” indicates the non-performed tests

Results of weight grasping tests are given in Table 1. Experimental snapshots of different grippers grasping a 50 g target are shown in Fig. 9c through f. We found that the gripper with softer actuators required lower air pressures to lift up the same weight of target. For example, gripper with actuator No. 1 could lift up a 70 g target with a pressure of 20 kPa, but it required 40 and 50 kPa for grippers with actuators No. 3, and No. 4, respectively, to lift up the same target. Actuator No. 2 performed the worst among the four actuators, and most of the failed grasps were caused by the unbalanced inflations of three actuators. We also found that the gripper with softer actuators generated less deformation on the paper container if we compared the container deformation in Fig. 9c, e, f. Significant deformation (Fig. 9d) was generated by the gripper with actuator No. 2 due to the individual differences of the actuators. In addition, we found that the influence of support material was not significant. Removing internal support material could slightly improve the grasping performance. We believe that this may be caused by the softer and more compliant property of the actuator without support material.

### Food material grasping

Grasping tests on the side dishes shown in Fig. 1b were conducted using actuator No. 1, and experimental snapshots are shown in Fig. 10. The weights are 25.3, 24.2, 31.6, and 23.2 g for ohitashi, hijiki, fried chicken, and salmon fish, respectively. Based on the experiments, it was easy to grasp and lift ohitashi and hijiki, but relatively hard to successfully lift up the salmon fish due to the irregular shape of the salmon fish.

### Durability tests

The durability tests were performed using the air control system shown in Fig. 11a. Two SMC valves (VQ110-5M-M5) were used to control the air input and output. A MOSFET was used to switch the valves on–off, and a pressure sensor (MIS-2500) was used to monitor the air pressure. The Arduino was programmed to realize a 0.2 Hz actuation frequency and count the actuation circles. We tested one actuator for type No. 1 at a pressure of 20 kPa, and two actuators for types No. 3 and No. 4 at their working pressures: 30 and 40 kPa for No. 3, and 50 and 60 kPa for No. 4. Test results for actuators of types No. 3 and No. 4 are shown in Fig. 11b. Actuator No. 1 was tested for more than 6000 circles and it does not seem to break or leak. Therefore, the results were not shown. We found that actuator No. 4 outperformed actuator No. 3 at low working pressure but underperformed at high working pressure. We believe that this is caused by the stiffer property of the AR-G1L material. We also investigated the influence of support material. We printed the actuator as two separate parts: the open chambers and a cover, as shown in Fig. 12a. By doing this, the internal surface of the chambers can be very smooth without support material. The chambers were then sealed by gluing the cover on it using a rubber targeted glue (ThreeBond 1521B). We tested two materials using this design. One is the TangoPlus material and the other is the TangoBlackPlus (Fig. 12a), which has the same hardness as TangoPlus but in black color. Test results are shown in Fig. 12b. Actuators with separate design and TangoPlus material reached more than 1000 circles at 30 kPa and around 500 circles at 40 kPa. Surprisingly, actuators made of TangoBlackPlus material realized more than 12,000 circles (only 6000 circles are shown in Fig. 12b) at 30 kPa and more than 4000 circles at 40 kPa.

## Conclusions

Grasping and handling a highly deformable object, such as a paper container filled with food material, is difficult due to the deformability and the complex contact conditions. Robotic gripper made of soft materials is able to adapt these difficulties and provides a possibility for handling such objects even without accurate control. In this study, we fabricated four types of pneumatic soft actuators using different materials and different fabrication processes. By comparing the performances among different type of actuators, we found that the 3D printed soft actuators had better linearity in the pressure-bending relationship and showed less individual differences thanks to the high printing resolution of the 3D printer. Actuator fabricated by two materials (actuator No. 2) showed nonlinear behaviors and significant individual differences due to the inhomogeneity and complex manual processes. Grasping tests showed that actuator made of softer materials required lower air pressure to grasp and lift the same weight of target. Meanwhile, softer actuators generated less deformation on the deformable target compared to harder actuators. Individual differences in actuator No. 2 resulted in uneven bending and further imposed unbalanced grasping forces on the target. Weight tests showed that actuator No. 1 could lift up to 90 g with a pressure of 20 kPa. Actuators No. 3 and No. 4 could lift up 70 and 80 g of targets with pressures of 40 and 60 kPa, respectively. Durability tests showed that 3D printed actuators, especially, the ones with support materials remaining inside chambers, had lower durability compared to the actuators fabricated by casting process. Without using support materials could slightly improve the grasping performance and significantly improve the durability of the actuator No. 3. Different 3D printable materials (TangoPlus or TangoBlackPlus) also affected the durability of the actuators. Apparently, at this moment, the traditional casted actuator (type No. 1) still outperformed the 3D printed actuators in both grasping performance and durability test despite the complex fabrication process. Nevertheless, along the rapid development of 3D printing technology, more printable soft materials and better performances are expected within a measurable period of time.

In this paper, only preliminary results were presented regarding soft gripper handling highly deformable objects. Many open questions are still remained untouched. Quantitative analysis on shape adaptability of soft gripper will be investigated together with optimized design of soft actuator structure. Durability tests on more 3D printed actuators and statistical results will be presented in the future. Improving the durability performance in the design point of view is another issue needs to be investigated.
